# Maxillary Third Molar Tooth Accidentally Displaced in Buccal Space: Report of Two Cases

**DOI:** 10.30476/DENTJODS.2020.87280.1250

**Published:** 2021-12

**Authors:** Kanj Hassan Wasfi, Hassib Nada Wayzani, Georges Aoun, Nicolas Antoine Berberi

**Affiliations:** 1 Postgraduate, Dept. of Oral and Maxillofacial Surgery, Dental Faculty, Lebanese University, Beirut, Lebanon; 2 Dental Faculty, Lebanese University, Beirut, Lebanon; 3 Dept. of Oral Medicine and Maxillofacial Radiology, Dental Faculty, Lebanese University, Beirut, Lebanon; 4 Dept. of Oral and Maxillofacial Surgery, Dental Faculty, Lebanese University, Beirut, Lebanon

**Keywords:** Maxillary, Third molar, Surgery, Complication, Buccal space, Fat pad

## Abstract

The extraction of retained and completely impacted third molars is one of the most common surgical procedures performed by dental practitioners with low rates of complications. The accidental displacement during the surgeries of the maxillary third molar into adjacent anatomical spaces is one of the most critical problems that can arise. The most common sites of migration during surgical interventions are the infratemporal fossa, the pterygomandibular space, the maxillary sinus, the buccal space, and the lateral pharyngeal space. In this paper, two cases in which a maxillary third molar accidentally was displaced into the buccal space are presented, the retrieval of the tooth via intra-oral approach is explained, and the anatomical spaces implications are discussed.

## Introduction

Post-operative complications can be observed during surgical extraction of third molars, such as uncountable bleeding, tooth root fracture, fracture of the tuberosity or the buccal bone, perforation of the sinus membrane, and prolapse of the buccal fat pad [ 1 ]. 

Few cases of accidental teeth displacement in direction of bordering anatomical areas such as the maxillary sinus [ 2 - 3 ], infra-temporal fossa [ 4 - 6 ], pterygoid-mandibular space [ 7 ], lateral pharyngeal space [ 8 ], and the buccal space [ 9 - 10 ] have rarely been reported. In the oral and maxillofacial region, many tissue spaces are inter-connected; consequently, a displaced tooth into one of these spaces can migrate to the others [ 10 ].

In this report, two cases of maxillary third molar that were moved accidentally toward the buccal space are described, and the extraction of the tooth via intraoral approach is explained. Moreover, this report reviews the anatomical spaces implications.

## Case Presentation 1

A 25 -year- old male was referred to our oral surgery clinic after unintentional movement of the third molar in the left side of the maxilla during surgical procedure under local analgesia. 

**Figure 1:**
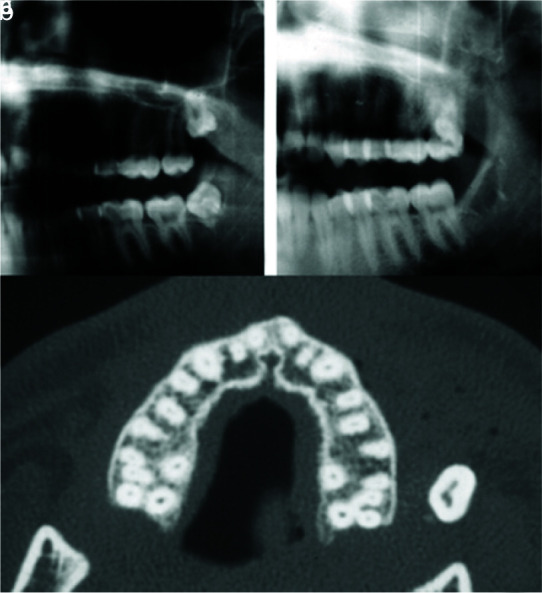
**a:** pre-operative panoramic x-ray showing the maxil-lary left impacted molar in place before the surgery. **b:** post-operative panoramic x-ray showing the maxillary left molar in a parallel position to the second molar. **c:** axial cut of the CT Scan showing localization of the displaced molar

The pre-operative panoramic X- ray shows the initial position of the third molar (Figure 1a). Intra oral palpation revealed a hard mass exists in the buccal space anterior to the coronoid process and the buccinator muscle was painful. A new panoramic X-ray radiograph showed that the third molar was displayed parallel to the second maxillary molar (Figure 1b). Axial images of computed tomography (CT) scan showed the position of the third molar in the left buccal space (Figure 1c). 

**Figure 2:**
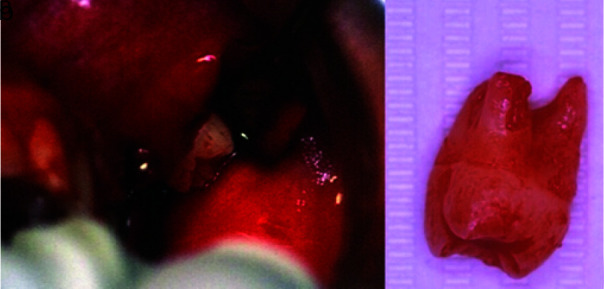
**a:** Intra-oral clinical view showing the molar coming out. **b:** The extracted tooth image that englobed the tooth

Surgery was accomplished under local anesthesia using (2% Articaine 1:100,000 adrenaline; 3M ESPE, Seefeld, Germany), tooth was reached after a submucosal incision in the buccal mucosa and was released from the surrounding tissue with a periosteal elevator (Figures 2a and b). The dissection of the fibrous connecting and the adipose tissues surrounding the tooth was the most difficult part of the surgery.

**Figure 3:**
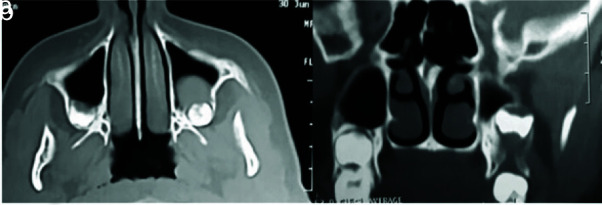
**a:** The axial view of the CT scan showing the position of the dental germ of the third molar and its relation to the maxillary sinus and the thin posterior cortical bone with a pathological image surrounding the tooth inside the sinus. **b:** Para-axial cut of the CT scan showing the high level of the third molar and the thin cortical buccal bone

After the tooth was retrieved and the mucosal tissues were secured with single simple interrupted sutures (Vicryl^®^ 3/0; Ethicon Johnson & Johnson, Somerville, NJ), non-steroid anti-inflammatory (Mefenamic acid 500 mg BID), analgesic (Acetaminophen 1000 mg in case of pain), and antibiotic (Amoxicillin 1g BID) drugs were prescribed for the patient. Keeping good oral hygiene (chlorhexidine digluconate 0.12 %) and the use of pack of ice were also recommended. The recovery period was without complications and he recuperated his mouth opening movements one week later (Figures 3a and b).

## Case Presentation 2

**Figure 4:**
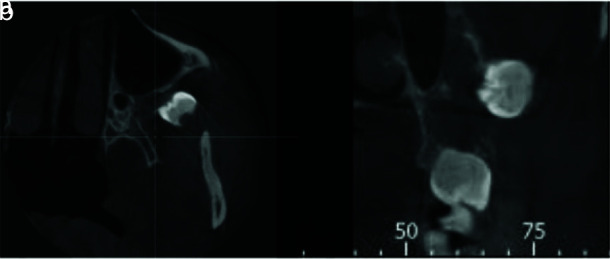
**a:** The axial view of a post-complication CT scan revealed the new position of the third molar in the buccal space. **b:** The para-axial cuts of the CT scan showing the movement in the buccal space of the tooth

A 16-year-old female was oriented to our surgical clinic by her dental practitioner. A surgical procedure was planned for an early removal of her left maxillary impacted molar for orthodontic reasons, but accidentally the tooth had been lost during the surgical procedure. Pre-operative axial and sagittal CT cuts showed a third molar in a very high position in relation with an inflamed maxillary sinus membrane and a dentigerous cyst. The new CT scan images showed that the molar was in the buccal space; it was jammed between the ramus and masseter and buccinator muscles, higher than the level the second molar for at least 2cm (Figures 4a and b).

**Figure 5:**
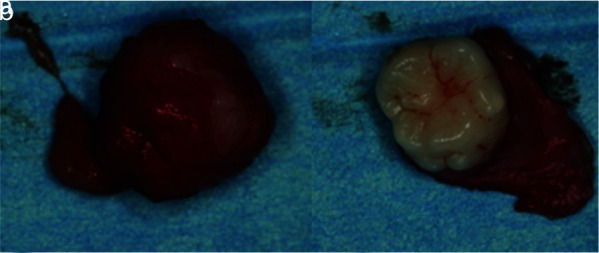
**a:** The removed germ encapsulated with a cyst. **b:** The crown surrounded by the cyst tissue

The intra-oral palpation revealed the deep position of the tooth in the buccal vestibule. Local analgesia was given to the patient followed by a submucosal incision the tooth was approached via blunt dissection using Metzenbaum scissors then with a tissue forceps the crown was reached and rotated and pulled out true the incision line. As in the first case, the difficulty faced during the surgical procedure was the dissection of the fibrous tissue and the tooth. After separating the third molar from the adipose tissue, it came out with the dentigerous cyst. Then, the mucosal tissues were secured with separate sutures like in the first case (Figures 5a and b). 

Postoperatively medication was used as in the first case. The recovery period was longer than the first case, which was most probably due to the age and the position of the displaced tooth.

## Discussion

Many complications associated with the surgical extraction of completely impacted maxillary third molars have been widely described in literature, such as osteitis, alveolar bone fracture, tooth fracture, tuberosity fracture, bleeding, oro-nasal communication, injury of adjacent teeth, infection, and accidentally displaced teeth. The most shared types of accidental displacement happen in the infratemporal fossa followed by the maxillary sinus. The use of elevators with excessive force associated with inadequate movements is mentioned as the most common errors related to iatrogenic displacements [ 11 - 12 ]. 

Inappropriate use of the dental elevators may provoke the tooth displacement due to a fracture of the buccal thin wall or the complete bone of the tuberosity, which is composed of cancellous bone surrounded by a thin cortical layer. When the tooth is on a very high position and the buccal bone is very thin, the risk of displacement of the impacted tooth in the buccal space is increased [ 9 ].

The maxillary third molar, is located very posteriorly on the dental arch; most often, it is located in the posterolateral part of the maxillary tuberosity and presents close relationships with the vasculo-nervous pedicle of the tuberosity, fascia of the buccinator and the infra-temporal fossa [ 3 ].

The maxillary third molars are limited by the buccal region laterally, the posterior palatal region medially (inside), the infra-temporal side of the maxilla and the infra-temporal fossa posteriorly, the maxillary arch anteriorly, and the maxillary sinus superiorly [ 10 ].

The fat bad, filled by adipose tissue, is on the buccal space and extends medially between the ramus and maxillary bone limited medially by the buccinator muscle, superficially by the deep cervical fascia and muscles of facial expression laterally and anteriorly, masseter muscle, mandible and the maxillary alveolar ridge, lateral and medial pterygoid muscles and the parotid gland posteriorly [ 9 ]. Buccal fat pad plays a major role in the muscular motions such those needed for the movements of the jaws [ 12 - 14 ]. The parotid duct, emerge from the gland and superficially to the masseter muscle opens on the inner surface of the cheek after piercing the buccinator muscle usually facing the second molar in the maxilla [ 11 ].

The position of the displaced tooth and its relation to other structures should be evaluated with a CT scan or a cone beam computed tomography (CBCT). Radiological images from CT scan or CBCT are required to localize the displaced tooth in two and three dimensions [ 15 ]. Radiological exams are suggested immediately before surgical procedure in order to localize the tooth and to ensure that, the displaced tooth would not affect the function of adjacent anatomical spaces [ 9 - 10 ]. 

The treatment decision for maxillary third molars is based on clinical and radiological information. A CBCT complete study is needed to evaluate the parameters that influence the surgical procedure [ 15 ].

Besides the surgical approach, the management of displaced maxillary third molar teeth is influenced by oral surgeon’s skill, experience, and adequate surgical tools. A conservative approach to remove tooth from the area ensures less post-operative complications.

Kocaelli *et al*. [ 9 ] reported a displacement of a third molar into the buccal space and concluded that the displacement was related to the luxation of third molar during surgical procedure and we agree with their conclusion. Ohba *et al*. [ 10 ] by using ortho-pantomograms (OPG) taken during the path of tooth’s migration demonstrated that maxillary third molar should be displaced laterally to the buccinator when displaced into the buccal space.

In our two cases, it has been confirmed, both clinically and radiologically, that teeth were displaced in the buccal space after applying a rotational forces from mesial to distal direction. To avoid surgical complications, careful attention to surgical details, including, a good interpretation of the radiological images, particular management of soft tissues, and a controlled force on the teeth and the hard tissue when applying surgical instruments must be respected. At the end of the treatment, patients showed a high satisfaction of the surgical procedures and no complications were reported. This case report was written after obtaining informed consent from the patient.

## Conclusion

Careful attention to surgical details, including, a good interpretation of the radiological images, particular management of soft tissues, and a controlled force on the teeth and the hard tissue should be regarded when applying surgical instruments to avoid such complications.

## Conflict of Interest

The authors do not have any financial interests, either directly or indirectly, in the products or information listed in this paper.
